# Should Men who have sex with Men be allowed to donate blood in Israel?

**DOI:** 10.1186/s13584-016-0123-2

**Published:** 2016-12-13

**Authors:** Gary Michael Ginsberg, Eilat Shinar, Eran Kopel, Daniel Chemtob

**Affiliations:** 1 Public Health Services, Ministry of Health, Yirmiahu Street 39, Jerusalem, 9446724 Israel; 2 Magen David Adom Blood Services, Ramat Gan, Israel; 3 Epidemiology Division, Ministry of Health, Jerusalem, Israel; 4 Department of Tuberculosis and AIDS, Ministry of Health, Jerusalem, Israel

**Keywords:** HIV, Men having Sex with Men, Transfusion Transmitted Infection, Blood Donations

## Abstract

**Background:**

The present permanent deferral policy in Israel for MSM was established in 1977 and was based on the previous (now outdated) USA Food and Drug Administration standards. This study analyses epidemiological data regarding blood donations among MSM, in order to estimate the risk for HIV transfusion transmitted infection (TTI) if the policy is changed to allow at-risk MSM to donate blood.

**Methods:**

An Excel based spreadsheet model integrated demographic, epidemiological data from the HIV National Register, laboratory, blood donation and testing data in order to calculate TTI due to false-negatives in known HIV+ donors, windows period donations, asymptomatic carriers and laboratory misclassification errors. A sensitivity analysis of our estimated TTIs for deferral periods for MSM was performed based on a literature review regarding this overall policy issue worldwide.

**Results:**

MSM in Israel have a considerably higher relative risk (RR) of both prevalence (115) and incidence (143) of being HIV+ than persons without a risk factor. Allowing MSM to donate blood, without any deferral period, will add an additional five HIV TTI cases over the next decade. Imposition of a 1 or 5 years deferral of abstinence will increase the number of HIV TTI cases only by 0.10 and 0.05 cases, respectively.

**Conclusion:**

A 1 year deferral period for blood donations from MSM in Israel is recommended.

## Background

There is an ongoing debate worldwide regarding permanent versus temporal deferral and receiving blood donations from persons with defined high risk behavior [1, 2], such as men who have sex with men (MSM) [3, 4]. The rationale and the benefits of such deferral/exclusion for MSM [5–10] have been questioned by lesbian, gay, bisexual, and transgender advocacy organizations seeking to change the current policy, in the light of technological advances in screening using Nucleic Acid Testing. This testing method enables a higher detection rate of contaminated blood by improving the tests sensitivity for the detection of causative agents of Transfusion Transmitted Infection (TTI) such as Human Immunodeficiency Virus (HIV), Hepatitis B (HBV) or Hepatitis C (HCV) and by reducing the length of the Window Period, during which detection is impossible [11, 12]. Injection drug users (IDU) are additional persons with defined high risk behavior for TTI with those viruses [13, 14].

In addition the Israeli population is composed of immigrants from 142 countries, as well as work seekers, some originate from specific countries with a Generalized HIV Epidemic, like Ethiopia. Due to high prevalence and incidence of mostly HIV and HBV, they are currently not allowed to donate blood. Although this was changed in april 2016 shortly after this papers acceptance.

The main reason for considering liberalizing blood donation policy for MSM is a response to the lesbian, gay, bisexual, and transgender advocacy organizations. The reason is not demand driven, since blood supply shortages are almost non-existent and the expected addition contribution if all MSM were allowed to donate is only around 1.5% of the blood supply. Any shortages can be made up by blood drives in the general not at-risk population.

Throughout the years since HIV detection, most countries have imposed an indefinite deferral period on MSM. However, since the introduction of Nucleic Acid Testing, some countries have instituted a permanent 1 years’ deferral period for MSM who wish to donate blood, with Canada allowing donations after a 5 years period of abstinence [15]. In December 2015 the US Food and Drug Administration announced its revised recommendations that changed the donor deferral policy for MSM to a 1-year deferral from last sexual contact [16]. In countries where MSM are not permanently deferred the sexual risk is assessed during the individual interview, conducted by a physician or nurse, at the blood donation site [17].

The present permanent deferral policy of MSM since 1977, of the Ministry of Health in Israel, was based on the previous (now outdated) FDA standards, and is reflected in the Donor Health Questionnaire and the personal interview, which are part of the screening process to potential blood donors. The questionnaire gives potential donors an option to self-defer from donating blood if they practice high risk sexual behavior (including MSM or receiving payment for sex), intravenous and non-prescribed drug usage or originating from countries with high prevalence and incidence of HIV, since 1977. The current deferral is permanent since 1977.

Every blood donation in Israel is tested for the detection of HIV-1, HIV-2, HBV, HCV and HTLV by chemiluminescent Immunoassay assay (ChlIA third generation, Prism, Abbott) and by an Individual NAT testing for HIV-1, HBV and HCV (Procleix Ultrio Assay, Grifols). Blood donation criteria and guidelines are continuously reviewed by the Advisory Committee to the Ministry of Health for Transfusion Medicine.

The current article combines demographic, epidemiological and laboratory data on MSM (and IDU) in order to calculate their relative risks (RR) of being HIV positive in addition to modeling an estimate of the absolute risk of HIV TTI as a result of enabling them to donate blood. This work was presented as evidence to an ad hoc committee nominated by the Director General of the Ministry of Health in early 2014 to examine blood donor policies in Israel.

## Methods

### Incidence and population data

HIV data from the Department of Tuberculosis and AIDS of the Ministry of Health, for the period 2005–2011, were integrated with population data from the Central Bureau of Statistics [18] in order to calculate incidence and prevalence rates among Israeli MSM aged 18–69 (the permitted age for blood donors) at high risk for HIV. These were compared with HIV incidence and prevalence rates among Israeli citizens who are not at high risk for HIV (ie: not MSM, IDU or Originating in countries with a Generalized Epidemic). The number of MSM of blood donation age (around 68,000) was based on a locally accepted estimate that 3% of males were MSM. Persons who were both MSM and IDU were excluded from the MSM group. The number of IDU was estimated to be 20,000 (Personal Communication: The Israeli anti-Drug Addiction Authority).

### Spreadsheet modeling

An Excel based spreadsheet model was used to integrate the demographic, epidemiological and laboratory data, together with data on blood donations and testing from the national Israeli Magen David Adom Blood Services (MDABS), in order to calculate the expected number of HIV positive cases that could be induced when changing the current deferral policy.

### Transmission pathways

We estimated the number of HIV transmissions from screened blood donations that could occur by the following four pathways (Table 1 lists the formulae for the calculations in detail):

**Table 1:** Calculation of magnitude of Transmission Pathways

The total expected number of transmission acquired HIV cases is = BLOOD x T x (A + B + C + D)
Where A is the number of infected donors, who donate despite knowing they are HIV+ A = POP x PREV x INFDON x (1-(1-((1-S_NAT_) x (1-S_EIA_))))
Where B is the number of infected donors during the windows period where neither the Nucleic Acid Test nor ELISA test can detect HIV. B = PSC x W_NAT_/(365.25 x YRS_PS_) PSC = POP x INC x YRS_PS_ x DON
Where C is the number of infected donors, who were asymptomatic and were not detected due to false-negative screening results resulting from the period where only the Nucleic Acid Test (but not the ELISA) can detect HIV (C_1_) and the period where both NAT and ELISA can detect HIV (C_2_). C = C_1_ + C_2_ C_1_ = PSC x (1-S_NAT_) *(W_NAT-_W_EIA_)/(365.25 x YRS_PS_)) C_2_ = PSC x (YRS_PS_-W_EIA_/365.25)/YRS_PS_ x (1-S_NAT_) x (1-S_EIA_)
Where D is the number of infected donors who are correctly detected but whose blood nevertheless is allowed to be donated due to laboratory misclassification errors. D = (PSC + (POP x PREV x INFDON)- 2 (A + B + C)) x LAB BLOOD = the average number of persons who become infected via transfusions from a single infected donor T = Probability that recipient of infected blood donation will become infected (%) POP = numbers aged 18–69 in group. DON = Percentage of people in group who donate blood (%) INFDON = Percentage of people who know they are HIV+ but nevertheless donate. PREV = Prevalence rate INC = Incidence rate per annum YRS_PS_ = Pre-symptomatic period: average length of time from infection to symptoms (years) based on weighted average of first-time to repeat donors over the 10 year period PSC = Number of pre-symptomatic donors B_1_ = number of infected donors during the window period of the Nucleic Acid Test. B_2_ = number of infected donors during period when the Nucleic Acid Test is working but EIS is still in window period S_NAT_ = Sensitivity of the Nucleic Acid Test W_NAT_ = Duration of the Nucleic Acid Test window period (days) S_EIA_ = Sensitivity of the anti-HIV test W_EIA_ = Duration of the anti-HIV window period (days) LAB = Laboratory misclassification error rate (%)

- False-negative screening results of people who knew they were HIV+ but nevertheless decided to donate blood.
- Blood donated during the “Window Periods” for anti-HIV antibodies testing of 15 days [1, 3, 19] (range 15–22 days [1]) and HIV Nucleic Acid Testing of 5.6 days [1, 11, 12] (range 5.0–6.2 days [1]).
- False negative screening results (ChlIA or Nucleic Acid Testing) from asymptomatic HIV cases, from the end of the window period till the median time of appearance of symptoms of 10 years [20]. These estimates are based on the sensitivity of HIV NAT testing of 0.9906 [12] (range 0.9761–0.9974) and anti-HIV testing of 0.9990 [3, 21], giving a combined sensitivity of 0.9999906.
- Results of human error, such as misclassification in the laboratory where positives were recorded as negatives or where infected blood had not been disposed of by mistake. We used a value of 0.026% (range 0.007% [3] – 0.05% [22]) based on the weighted average of three studies [3, 9, 22].

### Donation rates

In persons of blood donation age, there was a higher rate of overall donations among males than females (7.0% vs. 2.7% in females). We assumed that, if deselected, MSM will donate blood at the propensity as their gender specific counterparts in the population of people who are not a member of any high risk group (ie: not MSM, IDU or Originating in Countries with a Generalized HIV Epidemic).

### Transfusion infections

We next estimated the number of people infected with HIV from blood transfusions. According to data from MDABS each whole blood unit donated in Israel is divided to several components given to 2.5 persons on average. Using a 92.5% transmission rate [23] we applied the estimate that each contaminated donation will contaminate, on average, 2.31 persons.

### Sensitivity analyses

In addition, we performed a sensitivity analysis on our estimated TTIs based on the range of values (defined by the maximum and minimum of each parameter) for the test sensitivity, windows length, asymptomatic period and misclassification parameters.

### Effects of deferral

Since we did not have the necessary information to build a complex dynamic transmission model for HIV, we relied on estimates from the literature as to the effect of different deferral periods on donations from MSM, adjusted by the relative prevalence of MSM in the study countries to the Israeli situation, where an estimated 3% of males are MSM according to the advocacy groups of the gay community.

The reported effects of a 1 year deferral were from England and Wales with 3.5% MSM prevalence [3], Canada 4.5% [24] and the USA 6.0% [9]. The USA study [9] was supplemented by an additional study from England and Wales with a 3.5% prevalence [25] in order to estimate the effect of a 5 years deferral period. A further paper from England was not included as it was based on pre-Nucleic Acid Test detection technologies [26].

## Results

### Relative risks

MSM have considerably higher relative risks (RR) of prevalence (x115) and incidence (x143) of being HIV+ than NHR persons in the Israeli population (Table 2). Therefore, blood donors in Israel who are MSM are 115 times more likely to be infected with HIV+ than members of a non-high risk group.

**Table 2:** Epidemiological data by risk behavior

	MSM^a^	IDU^b^	No-Risk
			Group^c^
Population aged 18–69 in 2011	68,036	20,000	4,509,517
Prevalence HIV+	1,244	632	714
Prevalence/100,000 aged 18-69	1,828	3,160	15.8
Relative Risk of HIV prevalence	115	200	1
Annual Incident Cases	126	44	58
Incidence/100,000 aged 18–69 (2005–2012)	185	222	1.3
Relative Risk of HIV incidence	143	171	1

Notes:

^a^assuming MSM prevalence of 3% among males

^b^MSM who are also IDU are classified under IDU

^c^Rest of Population excluding Immigrants from countries with Generalized HIV Epidemic

The higher RR of HIV prevalence among MSM is reflected in the higher probabilities of a TTI resulting from an undetected infected donation (1:9,328 donations) compared with 1: 2,094,286 donations from persons not at a high risk of HIV (Table 3).

**Table 3:** Annual number of HIV+ TTI cases caused by blood transfusions from different risk behavior during the next decade

	MSM (a)	IDU (b)	No-Risk
			Group (c)
FN in known HIV+	0.00000007	0.00000002	0.0000001
Undetectable in Window Period	4.67	1.08	0.99
FN PS	0.07	0.02	0.02
Misclassification	0.25	0.06	0.02
Total TTI	4.99 (4.30-5.88)	1.16 (1.01-1.36)	1.03 (0.90-1.21)
No. of Donors	4,659	1,108	216,601
One TTI for every	9,328	10,718	2,094,286
Donations			
One year Deferral TTI	0.10 (0.07–0.19)	0.04 (0.03–0.08)	
Five year Deferral TTI	0.05 (0.03–0.08)	0.02 (0.01–0.03)	

Notes:

*FN* False - negative

*PS* Asymptomatic carriers

### Estimation of TTI cases

Our model predicted that if Israel continues with the current policy of only allowing members of NHR to donate blood, then around 1.03 HIV+ cases (95% CI: 0.90–1.21) will be transmitted during the next decade. The vast majority of these cases (96.1% or 0.99 cases) would arise from undetectable cases during the Window Period. A further 0.025 cases (2.4%) would arise from misclassification errors, which have grown in relative importance due to increasing sensitivity of tests and shortening of the windows detection periods [9]. An additional 0.016 cases (1.5%) will arise from false negative asymptomatic post-window period donations, whilst the number of false-negative symptomatic transmissions from donation from persons who already know they are HIV+ is negligible (Table 3).

Based on this data, allowing MSM to donate blood, without any deferral period, is likely to increase the number of HIV+ TTI cases by 4.99 (95% CI: 4.30 – 5.88) over the next decade, an almost six-fold increase in overall risk.

### Effect of deferral on TTI cases

Adjusted estimates (for MSM prevalence) of additional TTI in the case where MSM are allowed to donate blood after 1 year of reported abstinence, ranged from 6.4% [24] to 7.8% [3] to 15.7% [9] due to differences in parameters used in the three publications. Based on these estimates, the number of additional HIV+ TTI cases in Israel would increase by 0.10 (Range: 0.07–0.19) over a decade or one TTI every hundred years.

For a 5 year deferral period, the adjusted (for MSM prevalence) two estimates of the absolute increase in TTI were 2.8% [9] and 6.3% [25]. Based on this range, if policies were changed to allow MSM to donate blood after a 5 year self-reported abstinence rate, the number of HIV TTI would rise by around 0.05 (Range: 0.03–0.08) cases over a decade or one TTI every 200 years.

### Intravenous drug users

Just for comparison we performed a similar analysis of data regarding IDU in Israel, assuming IDU reporting behavior is the same as MSMs. We estimate that there will be 1.16 (95% CI: 1.01 –1.36) additional cases in the coming 10 years if IDU are allowed to donate and no-deferral is implemented, with 0.04 (Range 0.03–0.08) and 0.02 (0.01–0.03) additional cases if 1 or 5 years deferral from last exposure will be applied, respectively (Tables 2 and 3).

## Discussion

In view of the continuous pressure applied on decision makers in Israel to change the current indefinite deferral policy for MSM, we evaluated the additional risks of HIV TTI, using a self-built mathematical model based on inputs from the literature for scenarios where deferral for either a 5 or 1 year period after last sexual relation, or no- deferral is adopted.

Based on the epidemiological data in Israel, allowing MSM to donate blood, without any deferral period, will increase the number of HIV TTI cases by 4.99 from 1.03 to 6.02 cases over the next decade. Imposition of 1 and 5 year deferral rules will increase the number of HIV TTI cases among MSM by 0.10 (0.07–0.19) and 0.05 (0.03–0.08) cases respectively.

Unfortunately, it is impossible to predict future immigration patterns into Israel, whether from countries with high (e.g. Ethiopia) or low HIV prevalence (e.g. France) so our model made no attempt to adjust for future demographic changes among blood donors. Educational materials for donors and blood collection teams will be prepared ad-hoc, as required.

Our estimates of the number of TTI HIV cases are biased downwards for two major reasons:

1. The real windows period for anti-HIV testing, which accounts for some 77% of TTI cases in our model, can be in fact much longer than the 15 days that we used in our calculations. In fact, while these 15 days count only for the end of the eclipse period, the secoconversion period depends greatly on each individual, and can continue for several weeks [27]. Therefore, it is well accepted and practiced that when someone has a negative anti-HIV testing early after a potential HIV exposure, she/he should perform an additional anti-HIV testing 6 to 12 weeks after the exposure which was potentially at risk (and even later in some cases) [27, 28].
2. Our static model did not include any estimates of secondary transmissions generated by the initial infected blood recipient [3, 29].

Advocates of allowing members of high risk groups to donate blood use the following arguments:

- There will be an increase in the blood supply which will help other members of society.
- Since the current probabilities of being infected by blood transfusion are so absolutely low, the assumption is that potential recipients (i.e.: everyone in the society) do not see this as a risk. In economic jargon, there is a probabilistic threshold below which the marginal disutility of taking the risk is zero.

Changing to a temporal deferral policy may improve the reporting compliance of MSM who did not have sexual relations for the past 1 or 5 years, and by doing so, may in fact increase the safety of the blood donations. Many of the 68,000 MSM, not all of whom are high risk, would appreciate the change in policy and hence may be more likely to comply better with the new deferral policy.

By doing so, the safety of the national blood inventory might well increase. However one should notice that although the additional risk of TTI HIV among other people with risk behavior (i.e. about 20,000 IDU) is similar or even smaller (owing perhaps to their fewer numbers), no lobbies exist, to the best of our knowledge, whether in Israel or worldwide, for introducing changes in their permanent deferral policy.

The counter-arguments are:

- The expected increase of blood donations of currently deferred donors is negligible, adding only 2.1%, 1.0% [3, 9] and 0.3% [9, 22] additional donors with no deferral, 1 year and 5 years deferrals respectively.
- In Israel, like in other developed countries, a Patient Blood Management Program exists, causing a drop in the usage of blood units and components [30, 31] in times of peace. When an urgent need to increase the national blood inventory arises, it can be achieved by safer means such as increased recruitment of members of the large population group who are not at high risk, who have relatively lower donation propensities.
- Blood recipients in Israel (of a total population of over 8,000,000 persons) have the right to as safe a blood supply as possible. Hence they will be “less happy” (or in economic jargon: lose utility) if they perceive the blood supply as potentially more dangerous as a result of allowing at-risk groups to donate. A low-risk threshold does not exist. Even if it did exist, in an ex- post retrospective analysis one would have to take into account the suffering (dis-utility) caused to a small number of person, who will definitely be infected with HIV from transfusions.
- It is unethical to impose an additional risk for HIV on any population group (especially when they have not even been consulted) in order to decrease a feeling of discontent in other people who are currently considered to have higher risk, based on epidemiological data.
- Members of the non-risk-groups will feel good that others in the population (altruistic externality) are receiving as safe a blood supply as possible.
- Relaxing constraints totally on MSM and/or IDU donations will cause an increase in HIV+ blood donor related cases. Thus leading to the conclusion that there would be more benefits to the public from reducing the numbers of HIV+ MSM who donate blood, than from increasing the numbers of HIV+ MSM who may give blood [26].

While the AIDS-HIV registry enabled us to analyze relative risks for HIV by populations with risk behaviors, no such registries exist in Israel for HBV or HCV infections. Any change in policy needs to be accompanied by the establishment of a national monitoring program, to track rates of other infections (including other Transfusion-Transmitted Diseases) in the general population in order to assess risk factors in donors with HIV, HBV or HCV infections, and to study and evaluate these changes.

In deciding policy relating to blood donations one has to strike a balance between the safety of recipients, ensuring an adequate blood supply as well as societal/legal obligations to treat everyone fairly. Given that no transfusion is risk free, the question is what degree of risk is acceptable in order to meet the needs of recipients and society [32].

Lack of homogeneity of results of reported deferral periods caused us to just give a wide range of estimates for the effects of deferral periods on MSM. A Canadian study [24] reported that while the risk of implementing a 1 year deferral policy for MSM is very low, it can never be shown to be zero. They concluded that given today’s paradigm in blood safety, even a miniscule risk increment would be unjustified and undesirable. Another Canadian study reported that choosing a 1 year deferral period for MSM would almost certainly give rise to an incremental risk of TTI infection, and that such a policy would represent an unethical type of risk transfer from one social group to another, and would therefore be unacceptable [10]. Canada has since adapted a policy of allowing MSM to donate blood, after 5 years of abstinence, with a resultant worst-case estimate of one HIV contaminated unit every 1,072 years [33].

It should be noted that modeling studies indicate that adherence of potential blood donors to deferral policies is of major relevance, suggesting that good donor compliance may outweigh the negative effects on blood safety postulated for changing from permanent to temporary deferral periods for high risk sexual behaviors. The fact that a considerable percentage of donors are MSM - despite the permanent deferral policy [34] demonstrates the need to increase donor education and understanding [17]. It should be emphasized that our literature-based estimates of cases from MSM donors under a deferral system are based on a conservative estimate that does not take into account any possible gains resulting from less people giving false information about their MSM status as a consequence of instituting an MSM deferral period.

Whether or not to recommend the institution of a 1 year deferral period, as recommended lately by the FDA for the USA [16], or a 5 year period as in Canada [33] can be based on a value judgment as to whether or not 0.10 or 0.05 are subjectively acceptable increases in MSM TTI.

Based on the present model we recommend the institution of a 1 year deferral period for MSM to donate blood in Israel. However, we are aware of the fact that MSM are at a higher risk of other infections transmitted by blood (including Hepatitis B and C) than heterosexual men and women [13, 35, 36], so basing policy decisions solely on risks of TTI from just HIV in isolation is somewhat sub-optimal and inadequate. We therefore recommend that the suggested change in policy be accompanied by improving educational and other interventions with people with high risk behavior, upgrading the existing Nucleic Acid Testing and building a national hemo-vigilance and TTI monitoring system that will allow us to follow up the impact of such a policy change.

In addition, a recent publication from the USA showed that noncompliance with the MSM policy is evident and may be increasing compared to earlier data [37]. We join the authors’ recommendations that any change from the current policy requires close monitoring to determine whether it affects residual risk of HIV in the Israeli blood supply.

It should be also mentioned that there is an additional option of using an additional Nucleic Acid Test, 2 weeks post donation to the current Nucleic Acid Testing. If the additional Nucleic Acid Testing is negative then the Whole Blood unit which was held in quarantine can be released. However, this double- Nucleic Acid Testing protocol was considered to be unfeasible because of organizational constraints, stigmatization, loss of at least two major blood components and possible cost-effectiveness issues.

In the light of the elevated RR found in people with high risk behavior regarding HIV risks alone (as opposed to considering in addition hepatitis risks), we strongly recommend that if a change in the deferral policy for MSM is adopted, it must however be accompanied by the following steps:

1. Upgrading the current Nucleic Acid Test to a more advanced test generation, to allow earlier detection of HIV, HBV and HCV and the addition of HIV 2.
2. Implementing steps to improve education, attitude, knowledge and compliance of potential donors with high-risk behavior, with the deferral criteria.
3. Creating a national Hemo-vigilance program to collect data on blood donors and recipients, to monitor and ensure safer, best quality blood supply.
4. Finally, we recommend that any change in policy should be brought to the knowledge of the public, including analysis only. Ethical and societal issues are obviously related to such decisions, which often involve feelings of stigmatization and/or discrimination.

We note that the non-compliancy rate among MSM in Israel (i.e. MSM donate blood despite the restriction on accepting donations) is around 2.04% per annum, being of similar magnitude to that of 1.8% reported in Canada [38], 2.5% in the UK [39], 2.6% in the USA [37] and 4.5% in England [26]. Based on the UK and USA experiences, adoption of a 1 year deferral for donations from MSM is likely to reduce the non-compliancy rate by half [37, 39]. Furthermore, a non-compliancy rate as low as 0.2% has been attained in Australia [40], under a 1 year deferral regulation, by making the donors sign an extensive legal declaration that they are not MSM before donating.

## Conclusion

A 1 year deferral period for blood donations from MSM in Israel is recommended.

We trust that recommendation that is based on hard data provides an appropriate balance to the public’s fear of allowing more high risk people to donate and the perception of activist groups that they are being discriminated against by the current lifetime MSM deferral.

## Abbreviations

HBV: Hepatitis B Virus
HCV: Hepatitis C Virus
HIV: Human Immunodeficiency Virus
IDU: Injection Drug Users
MDABS: Magen David Adom Blood Services
MSM: Men who have sex with Men
RR: Relative Risk
TTI: Transfusion Transmitted Infection
